# The role of bacterial cyclic di-adenosine monophosphate in the host immune response

**DOI:** 10.3389/fmicb.2022.958133

**Published:** 2022-08-29

**Authors:** Xingqun Cheng, Jia Ning, Xin Xu, Xuedong Zhou

**Affiliations:** ^1^State Key Laboratory of Oral Diseases, National Clinical Research Center for Oral Diseases, West China Hospital of Stomatology, Sichuan University, Chengdu, China; ^2^The School and Hospital of Stomatology, Tianjin Medical University, Tianjin, China

**Keywords:** c-di-AMP, innate immune response, STING agonist, NF-κB pathway, inflammasome, autophagy, mucosal adjuvant, immunomodulation

## Abstract

Cyclic di-adenosine monophosphate (c-di-AMP) is a second messenger which is widely used in signal transduction in bacteria and archaea. c-di-AMP plays an important role in the regulation of bacterial physiological activities, such as the cell cycle, cell wall stability, environmental stress response, and biofilm formation. Moreover, c-di-AMP produced by pathogens can be recognized by host cells for the activation of innate immune responses. It can induce type I interferon (IFN) response in a stimulator of interferon genes (STING)-dependent manner, activate the nuclear factor kappa B (NF-κB) pathway, inflammasome, and host autophagy, and promote the production and secretion of cytokines. In addition, c-di-AMP is capable of triggering a host mucosal immune response as a mucosal adjuvant. Therefore, c-di-AMP is now considered to be a new pathogen-associated molecular pattern in host immunity and has become a promising target in bacterial/viral vaccine and drug research. In this review, we discussed the crosstalk between bacteria and host immunity mediated by c-di-AMP and addressed the role of c-di-AMP as a mucosal adjuvant in boosting evoked immune responses of subunit vaccines. The potential application of c-di-AMP in immunomodulation and immunotherapy was also discussed in this review.

## Introduction

Cyclic di-adenosine monophosphate (c-di-AMP) is a newly discovered signaling molecule after cyclic adenosine monophosphate (cAMP), guanosine tetraphosphate (ppGpp), and cyclic di-guanosine monophosphate (c-di-GMP). c-di-AMP was first identified in 2008 during the crystallization of a *Bacillus subtilis* (*B. subtilis)* DNA integrity scanning (DisA) protein (Witte et al., 2008). The *DisA_N* domain was characterized to have diadenylate cyclase (DAC) activity and could synthesize c-di-AMP using adenosine triphosphate (ATP) and adenosine diphosphate (ADP) as substrates. There are many other unknown functional domains around the DAC that presumably control the signal inputs and outputs of DisA (Witte et al., 2008). The DAC domain is widely distributed in Gram-positive and Gram-negative bacteria and archaea but not in eukaryotes. The Pfam protein database^1^ shows that there are 5407 proteins from 3991 species that contain the DAC domain, with a consensus sequence of 120 amino acids (Mistry et al., 2021). These species include not only most bacteria (not identified in the alpha, beta, and gamma proteobacteria) but also *Euryarchaeota.* This suggests an ancient origin of c-di-AMP, with critical roles in bacterial physiology that cannot be ignored.

Bacteria that lack the DAC domain, such as *Escherichia coli* (*E. coli)*, cannot synthesize c-di-AMP. Whereas *B. subtilis* has three DAC proteins, of which, DisA is regulated by the salt pressure response sigma factor SigM, and synthesizes 50% of c-di-AMP in early sporulation (Cao et al., 2002; Jervis et al., 2007). Since the regulatory networks of the three proteins in *B. subtilis* are completely different, they may play different roles under different environmental stimuli (Mehne et al., 2013). DAC activity in an alkaline pH is higher than that in an acidic environment and depends on divalent metal ions (e.g., Mg^2+^, Mn^2+^, and Co^2+^) (Bai et al., 2014). Proteins containing the DHH/DHHA1 domain, such as YybT (c-di-AMP phosphodiesterase) in *B. subtilis*, CnpB (cyclic nucleotide phosphodiesterase) in *Mycobacterium tuberculosis*, and Pde (phosphodiesterase) in *Streptococcus pneumoniae*, have phosphatase or phosphodiesterase activities, that can degrade c-di-AMP into pApA or AMP (Rao et al., 2010; Bai et al., 2014; Yang et al., 2014). The level of c-di-AMP increases considerably in the knockout strain for the gene encoding hydrolyase (Corrigan et al., 2011; Gall et al., 2022).

Cyclic di-adenosine monophosphate plays an important role in the regulation of bacterial physiological activities, such as the cell cycle, cell wall stability, environmental stress response, and biofilm formation. Moreover, c-di-AMP can be recognized by host cells for the activation of innate immune responses. In addition, c-di-AMP is capable of triggering a host mucosal immune response as a mucosal adjuvant. Therefore, c-di-AMP, a new pathogen-associated molecular pattern in host immunity, has become a promising target in vaccine and drug research.

## Cyclic di-adenosine monophosphate regulates physiological functions of bacteria

**FIGURE 1:**
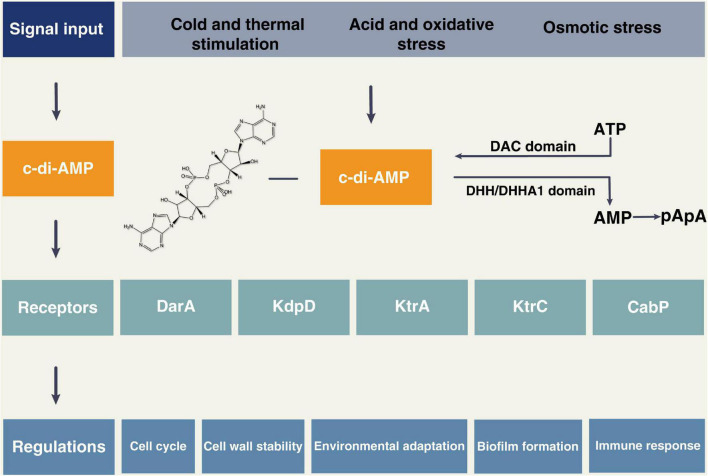
The cyclic di-adenosine monophosphate (c-di-AMP) signal system. c-di-AMP can be synthesized from ATP *via* proteins containing the DAC domain, and proteins containing DHH/DHHA1 domain can degrade c-di-AMP into AMP or pApA. The external stimuli can induce the production of c-di-AMP, which then binds to the receptors such as DarA, KdpD, KtrA, KtrC, and CabP. The c-di-AMP signaling pathway is involved in many bacterial physiological activities, such as the cell cycle, cell wall stability, environmental stress response, biofilm formation, and host immune response.

Cyclic di-adenosine monophosphate is involved in many essential cellular processes of bacteria, as shown in Figure 1 and Table 1. DisA scans the chromosome and pauses at sites of DNA lesions. Intracellular levels of c-di-AMP rise markedly at the onset of sporulation in a DisA-dependent manner. However, exposing sporulating cells to DNA-damaging agents can cause a decrease in the level of c-di-AMP, leading to a delay in sporulation, which can be controlled by the external supplementation of c-di-AMP. These results suggest that c-di-AMP acts as a critical secondary messenger in DNA integrity during sporulation (Boye, 2006). Both low and high levels of accumulation of c-di-AMP result in cell growth inhibition, suggesting a sensitive relationship between c-di-AMP homeostasis and cell growth (Mehne et al., 2013). Several studies revealed an essential role for c-di-AMP in peptidoglycan homeostasis and cell envelope stress response (Corrigan et al., 2011; Luo and Helmann, 2012; Wang et al., 2017). c-di-AMP fluctuates dynamically in coordination with Mg^2+^ and K^+^ levels, facilitating bacterial adaptation to osmotic stress (Zarrella et al., 2018; Quintana et al., 2019; Cereija et al., 2021; Wendel et al., 2022). Moreover, c-di-AMP could modulate bacterial acid stress, biofilm formation, and metabolism (Bowman et al., 2016; Townsley et al., 2018; Krüger et al., 2022).

**TABLE 1:** Summary of cyclic di-adenosine monophosphate (c-di-AMP) synthetases, catabolic enzymes, and related physiological functions in bacteria and archaea.

Microorganism	Synthetases	Catabolic enzymes	Receptors	Phenotypes	References
*Bacillus subtilis*	DisA, CdaA, CdaS	YybT, GdpP, PgpH	DarA, DarB, KtrA, KtrC, KimA	c-di-AMP increased: enhanced resistance to DNA damage and acid stress, inhibited biofilm formation, reduced excess c-di-AMP impair growth and virulence c-di-AMP: decreased DNA integrity, delayed sporulation, weakened cell wall, increased sensitivity to antibiotics, and impaired potassium ion channel system	Witte et al., 2008; Rao et al., 2010; Luo and Helmann, 2012; Townsley et al., 2018; Cereija et al., 2021; Krüger et al., 2022
*Listeria monocytogenes*	DacA (CdaA)	PdeA, PgpH, NrnA	LmPC, PstA, OpuC, CbpB	c-di-AMP increased: decreased growth, sensitivity toward acid stress and elevated osmotic stress, attenuated virulence in infection, enhanced IFN-β response in host cells c-di-AMP reduced: decreased growth, increased sensitivity to antibiotics, reduced cell wall stability, altered metabolic activity, and induced IFN-β response and cell pyroptosis	Woodward et al., 2010; Witte et al., 2013; Archer et al., 2014; Sureka et al., 2014; Huynh et al., 2016; Gibhardt et al., 2019; Gall et al., 2022
*Staphylococcus aureus*	DacA	GdpP, Pde2	KtrA, CpaA, PstA, KdpD, OpuC	c-di-AMP increased: smaller cell size, increased cross-linked peptidoglycan, enhanced resistance to β-lactam antibiotics and cell envelope stress, reduced carnitine uptake, increased acid sensitivity, impaired potassium ion channel system c-di-AMP reduced: slowed growth, increased salt tolerance, reduced methicillin resistance	Corrigan et al., 2011, 2013; Pozzi et al., 2012; Bowman et al., 2016; Schuster et al., 2016; Li et al., 2021
*Mycobacterium tuberculosis*	DacA DisA	Mtb PDE, CnpB		c-di-AMP increased: smaller cell size, attenuated virulence, induced IFN-β response, and increased induction of autophagy in host cells c-di-AMP reduced: slightly slower growth rate, increased virulence, decreased IFN-β response, reduced autophagy of host cells	Yang et al., 2014; Dey et al., 2015, 2017; Ning et al., 2021
*Lactococcus lactis*	CdaA	GdpP	LlPC, BusR, KupA, KupB	c-di-AMP increased: heat resistance, salt hypersensitivity, improved growth in response to penicillin G, increased osmoprotectants uptake c-di-AMP reduced: increased aspartate biosynthesis	Smith et al., 2012; Zhu et al., 2016; Choi et al., 2017; Quintana et al., 2019
*Mycobacterium smegmatis*	MsDisA	MsPDE	DarR	c-di-AMP increased: formed small colonies and enhanced intracellular C12-C20 fatty acid accumulation c-di-AMP reduced: cell death and reduced C12–C20 fatty acids production	Zhang et al., 2013; Tang et al., 2015
*Streptococcus pyogenes*	CdaA	GdpP, Pde2		c-di-AMP increased: impaired biogenesis of SpeB, increased biofilm formation, decreased virulence and increased antibiotic resistance c-di-AMP reduced: defect growth, inhibited biofilm formation, increased susceptibility toward environmental stressors	Cho and Kang, 2013; Fahmi et al., 2019
*Streptococcus pneumoniae*	DacA	Pde1, Pde2	CabP	c-di-AMP increased: impaired ability of long chain formation, decreased growth, and impaired potassium uptake, increased competitive ability c-di-AMP reduced: more susceptible to CSP	Bai et al., 2013, 2014; Zarrella et al., 2018, 2020
*Streptococcus mutans*	CdaA	PdeA, Pde2, (GdpP, DhhP)	CabPA, CabPB	c-di-AMP increased: increased biofilm formation c-di-AMP reduced: decreased growth rate, increased cell lysis, increased sensitivity to hydrogen peroxide and compromised competitiveness against *Streptococcus sanguinis*, enhanced polysaccharide synthesis	Cheng et al., 2016; Peng et al., 2016a,b; Konno et al., 2018
*Streptococcus suis* serotype 2	ssDacA	GdpP, Pde2		c-di-AMP increased: reduced growth, increased biofilm formation and reduced virulence	Du et al., 2014
Group B *Streptococcus*	DacA, NudP	CdnP	BusR	c-di-AMP increased: hyperosmotic susceptibility, increased induction of IFN-β response in host cells and decreased virulence	Andrade et al., 2016; Devaux et al., 2018
*Streptococcus mitis*	CdaA	GdpP, DhhP		c-di-AMP increased: slow growth, reduced rate of glucose metabolism, shorter chains, increased susceptibility to stress c-di-AMP reduced: longer chains, increased auto-aggregation but reduced biofilm formation	Rørvik et al., 2020, 2021
*Borrelia burgdorferi*	CdaA	DhhP		c-di-AMP is essential for cell growth and virulence, *dhhP* mutant was defective in induction of the σ(S) factor	Ye et al., 2014
*Enterococcus faecalis*	DisA	GdpP, DhhP		c-di-AMP reduced: inhibited growth, biofilm formation, and exopolysaccharide synthesis, sensitive to envelope-targeting antibiotics	Chen et al., 2018; Kundra et al., 2021
*Haloferax volcanii*	DacZ			c-di-AMP increased: cell death c-di-AMP reduced: impaired osmoregulation	Braun et al., 2019

Cheng et al. (2016) identified and characterized a DAC, CdaA (cyclic di-AMP synthase) in *Streptococcus mutans* (*S. mutans*), which is the primary etiological factor of human dental caries. Deletion of *cdaA* in *S. mutans* UA159 led to a decreased growth rate and increased cell lysis. The *S. mutans cdaA* mutant exhibited increased sensitivity to oxidative stress and compromised competitiveness against *Streptococcus sanguinis*. The global transcriptional data showed that several genomic islands, such as TnSmu1 (a conjugative transposon), TnSmu2 (a large transposon-like region), CRISPR1-Cas (clustered regularly interspaced short palindromic repeats/CRISPR-associated genes), and CRISPR2-Cas formed enriched interaction clusters in a *cdaA* mutant. Almost all of the upregulated genes formed a highly linked network, which indicated that a decrease in the c-di-AMP concentration in the mutant cells induced the expression of a set of genes with physiological functions (Cheng et al., 2016). Furthermore, c-di-AMP could regulate the expression of *glucosyltransferases* (*gtfs)* by interacting with CabPA (a c-di-AMP binding protein)/VicR (the response regulator in VicRK two-component signal transduction system) complex, modulating exopolysaccharide synthesis and biofilm formation of *S. mutans* (Peng et al., 2016a,b; Konno et al., 2018). All these studies indicate that c-di-AMP plays an important role in the regulation of bacterial physiological activities.

## Cyclic di-adenosine monophosphate modulates host innate immune response

Microbial challenges to the host trigger an array of defense processes through the activation of innate and adaptive immunity. Innate immunity is the first important line of defense against microbial invasion, especially for intracellular bacterial infection, and makes a critical contribution to many inflammatory conditions. The initiation of an inflammatory response relies on the recognition of a diverse range of pathogen-associated molecular patterns (PAMPs) and damage-associated molecular patterns (DAMPs) through pattern recognition receptors (PRRs) (Kumar et al., 2013). Different classes of PRRs include the Toll-like receptors (TLRs), C-type lectins, nucleotide-binding oligomerization domain-like receptors (NLRs), DNA sensors including those absent in melanoma 2 (AIM2), and RNA-sensing retinoic acid-inducible gene I-like helicases (Schroder and Tschopp, 2010). PRRs are not only expressed on the cell membrane but are also widely distributed in the endosomal membrane, lysosomal membrane, and cytoplasm. PRRs are found on both immune and non-immune cells and respond to PAMPs and DAMPs by initiating downstream signaling cascades, including the production and secretion of signaling mediators such as cytokines. Cytokines can be either pro- or anti-inflammatory and modulate the immune response, ensuring pathogen elimination, and removal of cellular debris, while also limiting excessive tissue damage (Seoane et al., 2020).

**FIGURE 2:**
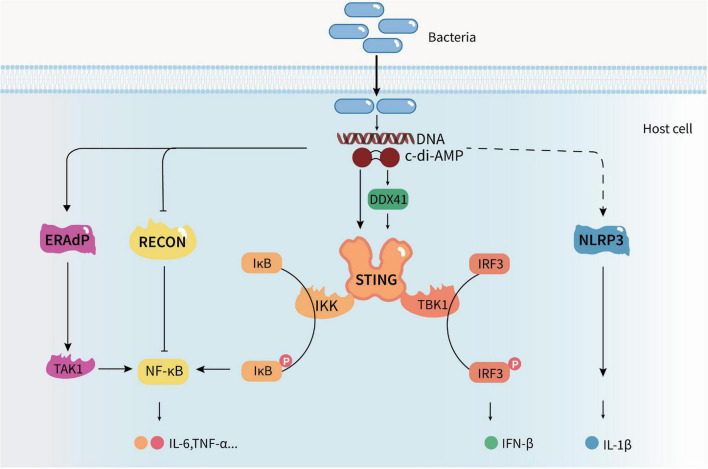
Cyclic di-adenosine monophosphate regulates the host’s innate immune response. c-di-AMP produced by pathogens can be recognized by host cells and activate host innate immune responses. It can induce a type I IFN response in a STING-dependent manner, trigger the nuclear factor kappa B (NF-κB) pathway by activating ERAdP or inhibiting RECON, induce NLRP3-dependent inflammasome response, and activate host cell autophagy, promoting the production and secretion of cytokines.

Cyclic di-adenosine monophosphate produced by pathogens can be recognized by host cells to activate immune responses (Figure 2). c-di-AMP may be recognized by multiple sensors/receptors in eukaryotic cells at the same time after bacterial infection. Therefore, they can precisely regulate the anti-infection status of the organism and affect the growth and spread of bacteria in the host. So far, four c-di-AMP sensors/receptors or adaptors have been found in eukaryotic cells: stimulator of interferon genes (STING) (Aliakbar Tehrani et al., 2021); RNA helicase DDX41 (D-E-A-D [aspartate–glutamate–alanine–aspartate]-box polypeptide 41) (Parvatiyar et al., 2012); reductase controlling The nuclear factor kappa B (NF-kB) (RECON) (McFarland et al., 2017); and endoplasmic reticulum membrane adaptor (ERAdP) (Xia et al., 2018). Hence, c-di-AMP is now considered to be a new PAMP in innate immunity (Dey et al., 2015). It can induce IFN response in a STING-dependent manner and activate the NF-κB pathway, inflammasome, and host autophagy.

### Cyclic di-adenosine monophosphate induces type I interferon response

The cytosolic surveillance pathway (CSP) is an important immune defense mechanism against bacterial or viral infection. The endoplasmic reticulum (ER)-associated protein STING encompasses a vital part of this immune cytosolic surveillance system. STING functions as a direct PRR of cyclic dinucleotides (CDNs) such as c-di-AMP and c-di-GMP. Originating from intracellular bacteria as an essential adaptor, STING indirectly detects cytosolic DNA through cyclic GMP-AMP (cGAMP) synthase (cGAS) (Aliakbar Tehrani et al., 2021; Guimarães et al., 2021). STING activation culminates in a robust inflammatory response associated with interferon regulatory factor 3 (IRF3) and the subsequent expression of IFN and other coregulated genes (Guimarães et al., 2021). The helicase DDX41 is also a PRR that senses both c-di-GMP and c-di-AMP, and DDX41 has a higher affinity for c-di-GMP than STING. DDX41 recognizes bacterial secondary messengers and forms a complex with STING to signal to TANK-binding kinase 1 (TBK1)-IRF3 activating a type I IFN immune response (Parvatiyar et al., 2012). Type I IFN is associated with efficient immune responses; it can activate host immune cells, such as macrophages and natural killer cells, and boost cell-autonomous defense mechanisms (Lee and Ashkar, 2018).

In Woodward et al. (2010) first identified that c-di-AMP secreted by intracellular *Listeria monocytogenes* triggers the host type I interferon response. The strong IFN-β-inducing ability of specific strains of *L. monocytogenes* is due to a genetic defect in *tetR* (tetracycline resistance transcriptional repressor) that results in the overexpression of *mdrT* (cholic acid efflux transporter) and a concomitant increase in c-di-AMP secretion (Yamamoto et al., 2012). Increased levels of c-di-AMP during intracellular infection led to the hyperactivation of the CSP. Conditional depletion of the *dacA* gene also caused increased IFN-β transcription and a concomitant increase in host cell pyroptosis, due to increased bacteriolysis and subsequent bacterial DNA release. Furthermore, c-di-AMP-activated STING-dependent type I IFN production inhibited cell-mediated immunity to *L. monocytogenes* (Archer et al., 2014). Gries et al. (2016) found that c-di-AMP released from the *Staphylococcus aureus* biofilm induced a macrophage type I IFN response. Moreover, a recent study demonstrated that thymidine metabolism disruption in *S. aureus* led to elevated c-di-AMP-mediated STING-dependent inflammation (Tang et al., 2022). These data suggest that c-di-AMP mediates the host innate immune response characterized by IFN-β release *via* the STING pathway.

Excess c-di-AMP secreted by a DAC-overexpressing *M. tuberculosis* strain could activate the interferon regulatory factor pathway with elevated levels of IFN-β in a STING-dependent manner, elicit increased macrophage autophagy and attenuate virulence in mice (Dey et al., 2015). The CnpB protein of *M. tuberculosis* hydrolyzes extracellular bacterial c-di-AMP and mutation of *cnpB* considerably enhances c-di-AMP secretion and accumulation. Infection with the Δ*cnpB* strain enhanced IFN-β expression in macrophages and exhibited significant inflammation attenuation. The infection resulted in less bacterial burden in the lungs and the spleen, thereby extending the survival of the mice. This indicated that the deletion of *cnpB* led to a reduction in the virulence associated with increased c-di-AMP levels (Yang et al., 2014; Dey et al., 2017). Group B *Streptococcus* also expresses an ectonucleotidase, *cdnP*, to mediate the hydrolysis of c-di-AMP. Inactivation of CdnP led to IFN-β overproduction, which is due to the cumulative effect of STING-dependent sensing of accumulated c-di-AMP outside the bacteria. Higher IFN-β levels *in vivo* increase bacterial killing by the host (Andrade et al., 2016). Pde2-deficient *S. pneumoniae* with elevated c-di-AMP drive aberrant innate immune responses from macrophages involving the hyperactivation of STING, excessive IFN-β expression, and rapid cytotoxicity (Wooten et al., 2020). The effect of the *Streptococcus suis* SntA (hemin-binding protein) on c-di-AMP is comparable with the activity of *cdnP* that dampened type I IFN response (Cabezas et al., 2022). These findings reveal that bacteria have developed a unique mechanism to inhibit STING activation and dampen the host cytosolic surveillance pathway. As bacteria can synthesize and degrade c-di-AMP, it suggests that c-di-AMP homeostasis plays a crucial role in governing pathogen infections.

Further studies have revealed that the endogenous ligand 2′,3′-cGAMP generated by cGAS had a higher binding affinity for STING compared with bacterial CDNs such as c-di-GMP, c-di-AMP, and 3′,3′-cGAMP (Gao et al., 2013). The extensive interactions and shape complementarity between asymmetric 2′,3′-cGAMP and the ligand-binding pocket make it the most preferred ligand for porcine STING. That geometry constraint limits the binding between symmetric 3′,3′-CDN and porcine STING (Cong et al., 2019). The ligand recognition and discrimination mechanism of porcine STING observed here expands our understanding of the activation of the CDN-STING pathway and its role in anti-pathogen defense. The 4′-thiomodified c-di-AMP analog, synthesized *via* the replacement of oxygen atoms with sulfur atoms at the 4′-position on the furanose ring of c-di-AMP, acted as a potent STING agonist and resulted in an enhanced immunostimulatory effect (Saito-Tarashima et al., 2021). Louie et al. (2020) explored the *in vivo* role of the STING pathway during bacterial pathogenesis and found that STING activation led to a reduced bacterial burden and correlated with the recruitment of monocytes to the intestines during *L. monocytogenes*-induced enterocolitis. This STING-mediated protective response was triggered by the c-di-AMP secretion of *L. monocytogenes*, while the disruption of type I IFN signaling did not recapitulate STING deficiency. These data suggest that STING signaling could provide pronounced contributions to immune responses at the initial site of infection in the mucosal barrier and that the activity of other immune sensors can compensate for STING function at later stages of infection.

Released type I IFN can bind to the IFN receptor, activating STATs (signal transducer and activator of transcription), mitogen-activated protein kinase (MAPK), and phosphatidylinositol 3-kinase (PI3K) signal pathways, regulating various physiological activities and initiating anti-pathogen responses. Mahmoud et al. (2019) demonstrated that c-di-AMP could specifically enhance the expression of additional inflammatory cytokines, namely, IL-6, CXCL2, CCL3, and CCL4, the mRNAs of which contain AU-rich elements (AREs) in the 3′ UTR that promote decay and repress translation. c-di-AMP was able to promote the phosphorylation of p38 MAPK as well as the induction of the ARE-binding protein TTP, both of which are components of a signaling pathway that modulate the expression of ARE-containing mRNAs at the post-transcriptional level. Pharmacological inhibition of p38 reduced the c-di-AMP-dependent release of induced cytokines, while TTP knockdown increased their release and mRNA stability (Mahmoud et al., 2019). These data propose that c-di-AMP can activate the p38 MAPK pathway *via* a post-transcriptional regulatory effect and subsequently enhance the expression of inflammatory cytokines.

Also worth noting is that c-di-AMP could significantly induce IFN-β response in dendritic cells. However, upon continuous cell stimulation, c-di-AMP induced downregulation of STING due to proteolytic degradation, leading to a decrease in IFN-β secretion (Rueckert et al., 2017). It suggests that bacteria-derived c-di-AMP can induce a host immune response, but the effect may be self-limited and discontinuous. It is indicated that the host has corresponding regulatory mechanisms to avoid an excessive c-di-AMP-induced immune response leading to immunopathologic damage.

### Cyclic di-adenosine monophosphate activates nuclear factor kappa B pathway

The nuclear factor kappa B is a ubiquitously expressed transcription factor that controls the expression of important regulatory genes that are responsible for various biological processes like immune responses, inflammation, cell proliferation, and apoptosis (Zinatizadeh et al., 2020). In the immune responses, NF-κB regulates the expression of many target genes that are playing critical roles in anti-infection and anti-inflammation processes such as immune recognition receptors, cytokines, antigen-presenting proteins, adhesion molecules, and chemokines.

Nuclear factor kappa B protein is located in the cytoplasm and can be activated by various cellular stimuli. Oxidoreductase RECON was revealed to negatively regulate NF-κB and STING activation. McFarland et al. (2017) identified that RECON functions as a cytosolic sensor for bacterial cyclic dinucleotides. High-affinity c-di-AMP binding inhibits RECON enzyme activity by simultaneously blocking the substrate and cosubstrate sites, inducing NF-κB activation and reducing bacterial survival. This suggests that c-di-AMP inhibition of RECON promotes a pro-inflammatory, anti-bacterial condition *via* STING and NF-κB pathways. However, other research by the same group demonstrated that *L. monocytogenes* secretion of c-di-AMP inhibits the enzymatic activity of RECON resulting in enhanced NF-κB activation and nitric oxide production, ultimately promoting cell-to-cell spread (McFarland et al., 2018). This finding is different from the previous recognization that c-di-AMP is capable of helping the host to clear the pathogenic bacterial infection. This indicates that despite c-di-AMP stimulation, the host immune response to infection depends on the type of pathogen.

The ERAdP acts as a direct sensor for c-di-AMP. c-di-AMP is bound to the C-terminal domain of ERAdP, which in turn leads to the dimerization of ERAdP, resulting in association with and activation of the kinase TAK1. TAK1 activation consequently induces the activation of the transcription factor NF-κB to induce the production of pro-inflammatory cytokines in innate immune cells (Xia et al., 2018). Moreover, ERAdP-deficient mice were highly susceptible to *L. monocytogenes* infection and exhibited reduced pro-inflammatory cytokines. Double knockout of ERAdP and TAK1 resulted in heightened susceptibility to *L. monocytogenes* infection. These data suggest that c-di-AMP is critical for controlling bacterial infection and can activate the NF-κB pathway through ERAdP mediation (Xia et al., 2018).

### Cyclic di-adenosine monophosphate activates inflammasome

The inflammasome is an essential component of the natural immune system (Lamkanfi and Dixit, 2014). The inflammasome is a family of multi-protein complexes comprising a cytosolic PRR, an adaptor protein called ASC (apoptosis-associated speck-like protein containing a caspase recruitment domain), and the protease caspase-1 (formed when the scaffolding PRR senses or binds to its activating stimuli). Following activation in inflammasomes, caspase-1 cleaves the inactive cytokine precursors pro-interleukin (IL)-1β and pro-IL-18, which subsequently leads to the secretion of their active forms (Liston and Masters, 2017). In addition, caspase-1 cleaves gasdermin D, which forms pores in the plasma membrane and, in many settings, can induce a rapid form of cell death called pyroptosis (Shi et al., 2015). Although several inflammasomes have been described (NLRP1b, NLRP3, NLRC4, and AIM2), the best-characterized inflammasome is formed by the NLR family pyrin domain-containing 3 (NLRP3) (Mangan et al., 2018). NLRP3 is mainly distributed in the cytosol and intracellular membrane of macrophages and neutrophils and is closely related to viruses and intracellular bacterial infection.

In previous studies, it was found that physiological levels of both c-di-AMP and c-di-GMP could induce the expression of caspase-1 and stimulate a robust secretion of IL-1β through the NLRP3 inflammasome (Abdul-Sater et al., 2013). However, this process was independent of STING. The response to c-di-GMP is dependent on the mobilization of potassium and calcium ions and is not associated with significant changes in mitochondrial potential or the generation of mitochondrial reactive oxygen species. It suggests that cyclic dinucleotides activate the NLRP3 inflammasome through a unique pathway that could have evolved to detect pervasive bacterial PAMPs associated with intracellular infections (Abdul-Sater et al., 2013). A recent study demonstrated that a combination adjuvant composed of c-di-AMP and a plant-derived nanoparticle significantly elicited an enhanced immune response to intradermal-injected vaccines in mice and pigs. The combination of adjuvant-induced robust activation of both NF-κB and IFN regulatory factor signaling pathways and the NLRP3 inflammasome, with increased expression of costimulatory molecules and secretion of TNF and IL-1β (Hernandez-Franco et al., 2021). Therefore, c-di-AMP activates an inflammasome that facilitates host anti-infection.

### Cyclic di-adenosine monophosphate activates host autophagy

Autophagy is a ubiquitous and fundamental eukaryotic pathway that has multiple effects on immunity. Autophagy contributes to the direct elimination of intracellular microbes, inflammation control, antigen presentation, lymphocyte homeostasis, and the secretion of immune mediators (Deretic et al., 2013). The microtubule-associated protein 1 (MAP1) light chain 3 (LC3) on the autophagosome membrane and isolation membranes is a reliable marker for the molecule of autophagosomes (Yoshimori, 2004).

Cyclic dinucleotides have been reported as potent autophagy inducers. Dey et al. (2015) first found that the expression of autophagosome membrane-specific markers (LC3-I and LC3-II) increased significantly in macrophages infected with recombinant *M. tuberculosis* overexpressing c-di-AMP synthetase. It is indicated that c-di-AMP activated autophagy, which resulted in the attenuation of the intracellular growth of bacteria in infected cells. Moretti et al. (2017) demonstrated that c-di-AMP in live Gram-positive bacteria served as a vita-PAMP, a specific class of PAMP that denotes microbial viability and elicits a commensurate innate response. c-di-AMP produced by bacteria was detected by the innate sensor STING, which then quickly mobilized interdependent pre-formed cell-autonomous responses, namely, ER stress, MTOR inactivation, and reticulophagy. ER stress induced macroautophagy/autophagy sequestered stressed ER membranes, resolved ER stress, and prevented apoptosis in response to live bacteria. This vita-PAMP-induced ER-phagy additionally orchestrates a type I IFN response by localizing ER-resident STING to autophagosomes (Moretti et al., 2017; Moretti and Blander, 2018). These findings identified stress-mediated ER-phagy as a cell-autonomous response mobilized by STING-dependent sensing of a specific vita-PAMP and elucidated how innate receptors engage multilayered homeostatic mechanisms to promote immunity and survival after infection. Ning et al. (2019) found that c-di-AMP evidently initiated autophagy with the elevated expression of genes associated with autophagy in macrophages. However, recombinant BCG vaccine formulated with c-di-AMP does not affect the level of LC3 (Ning et al., 2021). Another study revealed that the recombinant vaccine blocks autophagic flux by inhibiting p62 degradation, the level of which is negatively correlated with autophagy (Shen et al., 2022).

Autophagy is a precise regulatory process involving multiple genes. Factors such as nutrition deficiency and infection can induce autophagy, and the lack of inhibition of the autophagy signal is one of the important reasons for the immune escape of intracellular bacteria (Alharbi et al., 2021). Therefore, the activation of autophagy by increasing the level of c-di-AMP may assist the host in resisting infection by pathogenic microbes.

## Cyclic di-adenosine monophosphate triggers mucosal immune response as a mucosal adjuvant

The mucosal immune system is an immune response network composed of mucosal epithelium and secretion, mucosa-related lymphoid tissue, immunocompetent cells, and microflora that are widely distributed in the respiratory tract, gastrointestinal tract, urogenital tract, and their associated exocrine glands. Mucosal immunity is an immune response in local mucosal tissue induced by pathogens, food antigens, allergens, and other stimuli (Mettelman et al., 2022).

New effective adjuvants are necessary to boost and modulate evoked immune responses of subunit vaccines. Ebensen et al. (2011) found that c-di-AMP exerted strong adjuvant properties when delivered by the mucosal route. c-di-AMP is capable of stimulating macrophages as well as dendritic cells. Intranasal delivery of β-galactosidase adjuvanted with c-di-AMP to mice elicited significantly higher serum antigen-specific IgG and IgA titers compared with those in controls. The cellular immune responses were found to be strong with a balanced Th1/Th2/Th17 response pattern (Ebensen et al., 2011). After subcutaneous vaccination, recombinant vaccine adjuvanted with the STING-agonist c-di-AMP induced a significantly stronger Th1, IL-17, and IFNγ-producing cell response, outperforming other formulations, such as poly(I:C)/CpG and Tc52 (titanium salan complex) (Matos et al., 2017; Volckmar et al., 2019). Overexpressing DisA, the DAC of *M. tuberculosis*, in recombinant BCG increased IFN-β, IL-1, IL-2, IL-6, and IL-10 secretion, increased the expression of H3K4me3, an epigenetic marker of innate immune memory, and decreased bacterial burdens in the lungs and the spleen (Ning et al., 2019; Dey et al., 2020). The combination of the lipopeptide (*Mycoplasma fermentans*-derived)-based nanocarrier systems used for efficient antigen delivery across the mucosal barrier with c-di-AMP, allowed antigen dose reduction while maintaining vaccine efficacy against a respiratory challenge with a heterologous *Streptococcus pyogenes* strain (Schulze et al., 2017a). Nasal vaccination with BtaF (bacterial trimeric autotransporter adhesin) + c-di-AMP protects against intragastric challenge with *Brucella suis* by inducing local and systemic antibody responses with increased IL-2, IL-5, IL-17, and IFN-γ secretion, central memory CD4^+^ T cells and strong Th1 responses (Muñoz González et al., 2019). Oral administration of a genetically engineered *Lactococcus lactis* strain that simultaneously synthesizes the adjuvant c-di-AMP as well as a heterologous antigen is able to elicit a specific immune response, representing a new design strategy to develop a mucosal vaccine prototype (Quintana et al., 2018).

Vaccines adjuvanted with c-di-AMP induce strong humoral and cellular immune responses and protect against virus challenges. Recombinant hepatitis C virus vaccines adjuvanted with c-di-AMP evoked comparable neutralizing antibodies but a more robust cellular immune response, as revealed by strongly activated CD4^+^ T cells, compared with adjuvants using aluminum hydroxide/monophosphoryl lipid A (Alum/monophos./MPLA) and MF59 (an oil-in-water emulsion-based adjuvant) (Landi et al., 2017). c-di-AMP served as an adjuvant and provided efficient protection against influenza H5N1/H1N1 *via* the intranasal route in mice (Ebensen et al., 2017; Schulze et al., 2017b). In addition, c-di-AMP can also be combined with other adjuvants like alum, to produce a marked humoral and cellular immune response (Ebensen et al., 2019).

Furthermore, STING-agonist c-di-AMP coated on microneedles induced significantly higher IgG2a antibodies in serum as well as a higher level of Th1 cytokines (IFN-γ and IL-2) than that in a control group, suggesting that c-di-AMP could fulfill the role of adjuvants for microneedle-based skin allergen-specific immunotherapy (Shakya et al., 2018). To sum up, c-di-AMP promoted (i) effective local and systemic humoral immune responses, including protective hemagglutination inhibition titers; (ii) effective cellular immune responses, including multifunctional T-cell activity; (iii) induction of long-lasting immunity; and (iv) protection against bacterial or viral challenge (Ebensen et al., 2017; Schulze et al., 2017b). Hence, c-di-AMP, which has effective adjuvant properties, is a promising immunomodulator for the development of mucosal vaccines.

## Conclusion and outlook

As a microbial second messenger, c-di-AMP plays an important role in the regulation of bacterial physiological activities such as the cell cycle, cell wall stability, environmental stress response, and biofilm formation. Moreover, c-di-AMP produced by pathogens can be recognized by host cells and activate host innate immune responses. It can induce a type I IFN response in a STING-dependent manner, trigger the NF-κB pathway by activating ERAdP or inhibiting RECON, induce an NLRP3-dependent inflammasome response, and activate host cell autophagy, promoting the production and secretion of signaling mediators such as cytokines, thus modulating the host defense against infection. In addition, c-di-AMP is able to trigger a host mucosal immune response as a mucosal adjuvant. It can promote effective humoral and cellular immune responses, facilitating the induction of long-lasting immunity and protection against bacterial or viral challenges.

It is very important to gain more knowledge about the regulation of c-di-AMP production and degradation, and the role of c-di-AMP in bacterial physiological activities, environmental stress adaptation, and virulence. Also worth noting is that the role of c-di-AMP in archaea remains unclear, making it a valuable target for further study. Research on the effects of c-di-AMP on microbial physiology will lay a foundation for further studies regarding the immunomodulation activity of c-di-AMP. The roles of c-di-AMP-mediated bacteria and host interaction in the occurrence and development of diseases are yet unclear. In previous investigations, researchers found that c-di-AMP could regulate the gingival epithelial cytokine response and affect critical processes of gingival fibroblasts, playing a substantial role in the pathogenesis of the periodontal disease (Elmanfi et al., 2018, 2020; Onyedibe et al., 2021). More studies are needed to explore the regulatory effect of bacterial c-di-AMP on the immune microenvironment and the underlying mechanisms. Understanding the stimulation of the immune response by c-di-AMP may enable new approaches to achieve multilevel control of complex homeostatic processes in diseases and develop novel immunotherapies. In addition to acting as a mucosal adjuvant, c-di-AMP has gained attention in cancer immunotherapy. Vasiyani et al. (2021) demonstrated that c-di-AMP-activated and modulated the STING pathway to induce mitochondrial-mediated apoptosis in estrogen-receptor negative breast cancer cells. The combinatorial use of TLR9- and STING-agonist c-di-AMP induced innate and adaptive IFN-γ and exerted robust anti-tumor activities (Temizoz et al., 2022). Re-engineered BCG overexpressing c-di-AMP augmented trained immunity and exhibited improved efficacy against bladder cancer (Singh et al., 2022). In addition, small molecules have been designed to modulate the cyclic dinucleotide-cGAS-STING axis (Sintim et al., 2019), and inhibitors targeting *M. tuberculosis* cyclic dinucleotide phosphodiesterase have been identified in the regulation of the innate immune system (Karanja et al., 2021).

Also worth noting is that both c-di-AMP and c-di-GMP are critical bacterial second messengers regulating bacterial physiological activities and host immune responses. c-di-GMP is synthesized from two guanosine triphosphate (GTP) molecules by diguanylate cyclases (DGCs) and is degraded into pGpG by phosphodiesterases. While c-di-AMP is widely distributed in Gram-positive bacteria, c-di-GMP and its metabolizing enzymes have been identified in many Gram-negative bacteria such as *Pseudomonas aeruginosa*, *Caulobacter crescentus*, and *Escherichia coli*, and a few Gram-positive bacteria such as *B. subtilis* and *L. monocytogenes* (Ross et al., 1987; Kalia et al., 2013). c-di-GMP mainly regulates the bacterial motility-to-sessility transition, biofilm formation, and virulence factor production (Randall et al., 2022). Both c-di-AMP and c-di-GMP can trigger type I interferon response. However, c-di-GMP has a higher affinity for DDX41 compared with that STING (Burdette et al., 2011; Parvatiyar et al., 2012; Li et al., 2019). Intranasal c-di-GMP administration did not cause strong inflammatory responses or lung injury but enhanced type II (IFN-γ)/III IFN (IFN-λ) production and Th1, Th2, and Th17 responses with the production of polarizing cytokines, namely, IL-6, IL-12, IL-13, IL-23, and TGF-β in mice (Yan et al., 2009; Blaauboer et al., 2015). Moreover, c-di-AMP and c-di-GMP activate the NLRP3 inflammasome through a unique pathway (Abdul-Sater et al., 2013). Therefore, cyclic nucleotide signaling molecules act in a highly specific manner forming a multilayered signaling scaffold. More studies are needed to elucidate the function of cyclic nucleotides, such as c-di-AMP and c-di-GMP, in bacterial physiology as well as in the host immune system. The associations among these cyclic nucleotides also need further exploration and may provide novel targets for preventing infections.

In conclusion, c-di-AMP plays a major role in host immune responses and has become a promising target in vaccine adjuvants and immunotherapy.

## Author contributions

XC and JN drafted the manuscript. XX and XZ edited and added valuable insights to the manuscript. All authors approved the final manuscript and agreed to be accountable for all aspects of the work.

## Footnotes

1
http://pfam.xfam.org/family/PF02457.19#tabview=tab1

## Abbreviations

c-di-AMP: cyclic di-adenosine monophosphate
cAMP: cyclic adenosine monophosphate
c-di-GMP: cyclic di-guanosine monophosphate
cGAMP: cyclic GMP-AMP
ppGpp: guanosine tetraphosphate
DAC: diadenylate cyclase
PAMPs: pathogen-associated molecular patterns
DAMPs: damage-associated molecular patterns
PRRs: pattern recognition receptors
STING: stimulator of interferon genes
DDX41: D-E-A-D [aspartate–glutamate–alanine–aspartate]-box polypeptide 41
RECON: reductase controlling NF-κB
ERAdP: endoplasmic reticulum membrane adaptor
CSP: cytosolic surveillance pathway
CDNs: cyclic dinucleotides
IFN: type I interferon
MAPK: mitogen-activated protein kinase
NF-κB: nuclear factor kappa B
NLRP3: nucleotide oligomerization domain-like receptor family pyrin domain-containing 3.
